# New Licensing Rules of the L.C.C.

**Published:** 1920-10-30

**Authors:** 


### New Licensing Rules of the L.C.C.

From January 1, 1920, the rules which govern establishments for massage or special treatment in the County of London are to be considerably tightened up. Under Section 23 of the London County Council (General Powers) Act, 1920, Part IV., the Council directs that after the above date no person may carry on in the County of London without a licence any establishment for the reception or treatment of persons requiring massage, manicure, chiropody, light, electric, vapour, or other baths, or other similar treatment. These provisions will affect numerous nursing homes. They do not apply to certain hospitals, infirmaries, Government, municipal, and other institutions, of a like character, philanthropic institutions, or establishments carried on by qualified medical practitioners. Hairdressers' shops are also exempted under certain conditions.

Licences must be applied for before November 19. The fee for a new licence for persons not already registered, to run till March 1922, is £2 2s., that for renewal of licence £1 1s. The Act gives the authorities the right to enter any premises used for the above purposes and inspect the entries in the records required to be kept. A highly valuable part of the new regulations consists in powers to make by-laws as to the technical qualifications to be possessed by persons administering massage or other curative treatment in the licensed premises. Very heavy penalties are imposed on persons contravening the regulations. Fines up to the amount of £50 or imprisonment up to a term of three months may be inflicted for carrying on homes without a licence or in other ways infringing the regulations. It may be noted that a new licence is required in cases in which any change occurs in the constitution of a partnership, or when a business is transferred to a limited liability company, or when a business is transferred from one person, society, etc., to another. The trade name or style or title of an establishment may not be changed until the licence has been returned to the Council for endorsement. And in cases where other premises are to be occupied an additional licence for which the nominal fee of 1s. will be charged must be obtained. One more precaution. Should the nursing home or other establishment introduce any new form of treatment not specified in the original licence the Council must be notified and the licence returned for endorsement. Too much care cannot be taken to follow out faithfully the regulations now intended to be put in force. They constitute a tower of strength and protection for all carrying on useful work for the public of this description.

